# Physiological adaptations and practical efficacy of different blood flow restriction resistance training modes in athletic populations

**DOI:** 10.3389/fphys.2025.1683442

**Published:** 2025-10-29

**Authors:** Chuang He, Dinghuang Zhu, Yunzhou Hu

**Affiliations:** ^1^ Department of Physical Education, Xi’an Binhe School, Xi’an, China; ^2^ School of Athletic Performance, Shanghai University of Sport, Shanghai, China

**Keywords:** blood flow restriction, resistance training, muscle hypertrophy, skeletal muscle, athletes

## Abstract

Blood flow restriction resistance training enhances athletic adaptations via distinct mechano-metabolic pathways. This review synthesizes evidence comparing three blood flow restriction resistance training modalities: Low-load resistance training with blood flow restriction (using 20%–30% of one-repetition maximum) prioritizes metabolic stress (lactate and hydrogen ion accumulation, cellular swelling), activating growth hormone (GH)/insulin-like growth factor 1 (IGF-1)/mechanistic target of rapamycin (mTOR) pathways to promote type I muscle fiber hypertrophy, making it suitable for joint-sparing rehabilitation scenarios. Supplemental blood flow restriction resistance training programs combine high-load tension (utilizing 75%–90% of one-repetition maximum) with additional blood flow restriction to produce an acute synergistic effect. This method enhances the recruitment of type IIa/x muscle fibers and prolongs mTOR phosphorylation. Combined blood flow restriction resistance training employs alternating cycles of high-load phases (70%–85% 1RM) and blood flow restriction phases (hypoxia-inducible factor 1-alpha (HIF-1α)-mediated angiogenesis), optimizing phosphocreatine resynthesis and neural drive to achieve specialization of type IIx muscle fibers. Periodized application requires matching modalities with training phases: combined blood flow restriction training for hypertrophy during the preparatory phase, supplemental blood flow restriction training for strength maintenance during the competitive phase, and low-load resistance training with blood flow restriction for active recovery. This mechanistic framework supports evidence-based blood flow restriction resistance training programming to maximize athletic adaptations while mitigating injury risk.

## 1 Introduction

Blood Flow Restriction Training (BFRT) applies external pressure to the proximal limbs to partially restrict arterial inflow and fully occlude venous return (Centner et al., 2019). First introduced by Yoshiaki Sato in 1983, the method alters local hemodynamics to induce metabolite accumulation, cellular swelling, and hypoxia, thereby activating high-threshold motor units and stimulating muscle adaptation under low mechanical loads (Fahs et al., 2015). BFRT resistance enhances skeletal muscle strength with distinctive practical value. Compared to traditional high-load resistance training, combining BFRT with low-intensity loads (typically 20%–50% 1RM) yields similar hypertrophy and strength gains while minimizing joint stress—making it particularly suitable for athlete rehabilitation and in-season strength maintenance (Huang and Park, 2024; Pignanelli et al., 2021).

Based on the combination of training load and blood flow restriction, BFRT resistance training modalities described in the literature can be categorized into three main types: low-intensity resistance training with blood flow restriction (BFR-LIRT) (Korkmaz et al., 2022), high-intensity resistance training supplemented with BFRT (HIRT-BFRT, involving high-load resistance exercises followed by low-load BFRT) (Luebbers et al., 2014), and integrated high-intensity resistance training combined with BFRT (HIRT + BFRT, characterized by periodic alternation between high- and low-load sessions, e.g., one high-load session plus two low-load BFRT sessions per week) (Sarfabadi et al., 2023). Existing evidence suggests that these BFRT resistance modalities exert differential effects on skeletal muscle strength (Scott et al., 2023). Over medium-to long-term interventions, all three have been shown to increase muscle cross-sectional area (CSA) and enhance maximal strength.

To evaluate BFRT resistance effects in athletes, we systematically searched CNKI, PubMed, and Web of Science, including only studies involving athletes with structured BFRT interventions and control groups. From 412 initially identified articles, 14 met the inclusion criteria. This narrative review compares the physiological mechanisms and practical efficacy of these BFRT resistance modalities and offers evidence-based rationale for their use across rehabilitation, competitive, and preparatory phases. We outline underlying neuromuscular, metabolic, and molecular mechanisms; evaluate empirical effectiveness; and discuss implications for individualized training and future research.

## 2 Physiological mechanisms of BFRT

Blood flow restriction resistance training significantly enhances athletes’ muscle strength, and this improvement is strongly correlated with an increase in the CSA (Wilk et al., 2018). Exercise-induced metabolic stress and mechanical tension are key drivers of muscle hypertrophy, and these mechanisms act synergistically to collectively act to significantly elevate the rate of protein synthesis (Li et al., 2024). Traditional resistance training primarily relies on mechanical tension to stimulate myofibrillar protein synthesis through mechanotransduction pathways. In contrast, blood flow restriction training uniquely induces significant metabolic stress, leading to comparable muscle growth but with lighter loads than those required for HLRT (Dong et al., 2025). However, the relative contribution of these two primary mechanisms varies considerably depending on the specific BFRT modality employed. To elucidate these distinctions, the following sections are organized according to the three predominant BFRT modalities—BFR-LIRT, S-BFRRT, and C-BFRRT—as this framework most clearly delineates their unique mechanistic signatures and adaptive outcomes.

### 2.1 BFR-LIRT: metabolic stress-dominated adaptation

#### 2.1.1 Metabolite induced fatigue

During BFR-LIRT, metabolites such as lactate, H^+^, ATP, and inorganic phosphate accumulate locally within the limb owing to impaired venous outflow (Loenneke et al., 2012). These metabolites impair excitation-contraction coupling and enhance the recruitment of type II muscle fibers (Wernbom and Aagaard, 2020). Accumulating metabolic stress reduces contraction velocity while enhancing muscle activation, a phenomenon that promotes anabolic signaling (Takarada et al., 2000). Additionally, accumulated metabolites activate group III-IV afferents, which enhance motor unit recruitment through gamma-loop feedback to sustain force production. This activation also increases synaptic activity within the central nervous system, thereby elevating perceived exertion (RPE) (Amann et al., 2015). At the molecular level, lactate stimulates muscle hypertrophy by inhibiting histone deacetylase activity (Pearson and Hussain, 2015). Additionally, H^+^ accumulation redistributes the workload to type I fibers through altered calcium sensitivity (Hostrup et al., 2021), promoting preferential hypertrophy of slow-twitch fibers (Bjornsen and Wernbom, 2019b). While existing evidence highlights metabolic stress as a key driver of BFRT-induced adaptations, the direct role of metabolites in mediating muscle hypertrophy requires further investigation to be fully understood. Further research is required to elucidate the fiber-type-specific molecular mechanisms underlying these processes.

#### 2.1.2 Cell swelling

BFR-LIRT induces marked cellular swelling, primarily involving fluid retention owing to venous occlusion (Cleary et al., 2022). Acute increases in muscle thickness result from plasma-to-cell fluid shifts driven by osmotic gradients. Exercise-induced swelling is associated with multiple factors, including local hypoxia, metabolite accumulation, and reactive hyperemia, which collectively enhance type II fiber recruitment (Mouser et al., 2017). BFRT-induced swelling produces similar hypertrophic effects to HLRT (Alvarez et al., 2020). At the molecular level, swelling activates mechanosensitive pathways (e.g., mTORC1), and fluid-induced cytoskeletal stress triggers anabolic signaling cascades. However, passive swelling alone fails to upregulate mTORC1 expression (Nyakayiru et al., 2019), indicating that mechano-metabolic synergy is essential. Chronic BFRT counteracts disuse atrophy by sustaining exercise-induced cellular swelling, thereby creating a favorable anabolic environment that helps maintain net protein balance even under low-load resistance stimuli (Manimmanakorn et al., 2013; Martín-Hernández et al., 2013). This mechanistic insight into how BFRT preserves muscle mass underlies its growing application in both rehabilitation and athletic training settings.

#### 2.1.3 Mechanical tension

**FIGURE 1:**
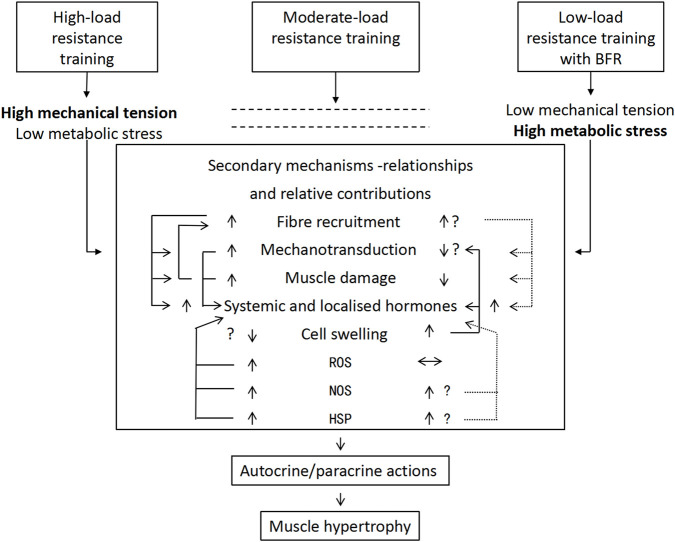
Mechanism of resistance training load/pattern on muscle hypertrophy (Pearson and Hussain, 2015). Note: The relative contributions of mechanical tension and metabolic stress in mediating muscle hypertrophy, depending on the training load and/or modality. The arrows highlight the potential degrees of activation of the resultant intermediate secondary mechanisms and their possible relationships. Vertical arrows (↕) represent higher/lower degrees of activation; horizontal arrows (↔) represent no effect; interconnecting arrows represent potential relationships between secondary mechanisms; and dotted interconnecting arrows indicate equivocal relationships. HSP, heat shock proteins; NOS, nitric oxide synthase; ROS, reactive oxygen species.

Existing evidence indicates that mechanical tension mediates muscle hypertrophy during moderate-to-HLRT. Evidence has confirmed that mechanical load prevents denervation atrophy (Goldberg et al., 1975). This confirms the crucial role of mechanical stimuli in muscle development. Subsequent animal (Spangenburg, 2005) and human studies (Schoenfeld, 2013b) have validated this dose-response relationship. At the molecular level, mechanical tension promotes muscle growth through multiple pathways, including the activation of mechanotransduction (e.g., the mTOR pathway), upregulation of local growth factor expression, induction of moderate muscle microdamage, modulation of NOS, HSP, and ROS production, and promotion of selective fast-twitch fiber hypertrophy (Cook et al., 2013). These mechanisms ultimately activate satellite cells and increase the rate of protein synthesis (Wernbom and Aagaard, 2020). BFR-LIRT generates less mechanical tension but compensates through metabolic stress, achieving similar hypertrophic effects (Oliveira and Campos, 2020). Training modes differ significantly in their tension/stress profiles: HLRT is characterized by high tension and low stress, whereas moderate-load training achieves a balance between the two. This dose-response variation implies distinct strategies, as moderate conventional training, HLRT, and low-load BFRT induce muscle hypertrophy through distinct mechanistic pathways. These findings provide a scientific foundation for the development of personalized training strategies. Figure 1 summarizes the conceptual interplay between mechanical tension and metabolic stress across different training modalities. It illustrates how HLRT primarily activates mechanisms via high mechanical tension (e.g., mechanotransduction, microdamage), whereas BFRT augments metabolic stress pathways (e.g., cellular swelling, ROS signaling) to achieve hypertrophy even at low loads. This model provides a visual framework for understanding the distinct yet synergistic mechanistic emphasis of the BFRT modalities discussed herein.

**FIGURE 2:**
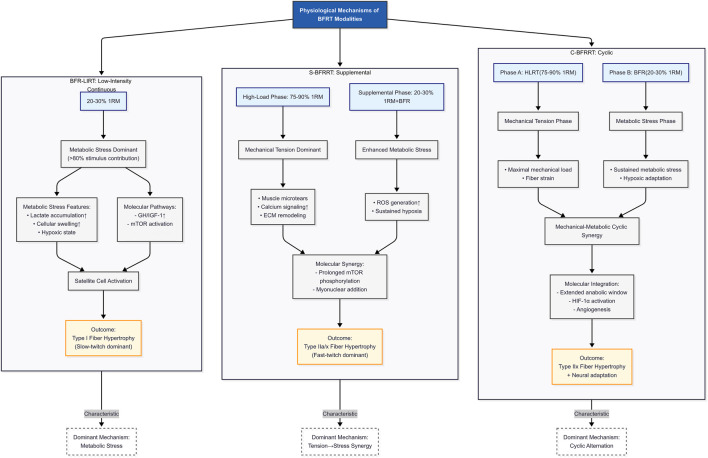
Comparative physiological signatures of BFRT modalities: Postulated fiber-type-specific hypertrophy mechanisms. Note: 1RM, One-repetition maximum; ECM, Extracellular matrix; ROS, Reactive oxygen species; HIF-1α, Hypoxia-inducible factor 1-alpha; mTOR, Mechanistic target of rapamycin; type I, Slow-twitch oxidative fibers (endurance-oriented); Type IIa, Fast-twitch oxidative-glycolytic fibers; Type IIx, Fast-twitch glycolytic fibers (power-oriented).

Through a systematic analysis of existing studies, significant differences exist in the dominant mechanisms by which different BFR resistance training modes promote muscle strength gains (see Figure 2). Currently, blood flow restriction resistance training is primarily divided into three core modes: the first modality, BFR-LIRT, operates primarily through metabolic pathways, utilizing 20%–30% 1RM loads. The resulting GH secretion (3–5× baseline) and type I fiber activation are characteristic responses to BFR-LIRT, suggesting that this modality is particularly well-suited for rehabilitation settings and off-season strength maintenance, where high mechanical loads are contraindicated (Hanke et al., 2020).

### 2.2 S-BFRRT: sequential mechano-metabolic synergy

S-BFRRT combines high-load mechanical tension (75%–90% 1RM) with supplemental blood flow restriction (20%–30% 1RM) in a sequential manner, creating synergistic effects through metabolic and mechanical stimuli. The initial high-intensity phase induces sarcomere microtears and calcium-dependent mTOR activation, priming the muscle for metabolic amplification (Schoenfeld, 2013a). Subsequent BFR supplementation traps reactive oxygen species (ROS) and lactate in damaged fibers, prolonging mTOR phosphorylation for >48 h through a markedly enhanced activation of ribosomal S6K1 compared to BFR-LIRT alone (Oliveira and Campos, 2020). This process enhances satellite cell recruitment into type IIa/x fibers, resulting in significantly greater hypertrophy compared to traditional high-load training (Wilk et al., 2018).

### 2.3 C-BFRRT: combined training - spatiotemporal collaborative adaptation

C-BFRRT establishes cross-adaptive loops through periodized alternation of high-intensity resistance training (HLRT; 70%–85% 1RM) and BFR phases (20%–30% 1RM). Phase A upregulates PGC-1α pathways to enhance mitochondrial biogenesis in type IIx fibers, While Phase B activates HIF-1α-mediated angiogenesis, it significantly increases capillary density (Li et al., 2024). Crucially, metabolic phases clear interleukin-6 via muscle-pump-driven lymphatic drainage, a recovery mechanism absent in BFR-LIRT (Korkmaz et al., 2022). This rapid clearance is thought to be beneficial because, while IL-6 is a multifunctional cytokine released during exercise, its prolonged elevation can contribute to sustained inflammatory signaling and fatigue (Zhu et al., 2025). By promoting its efficient removal, C-BFRRT may potentially accelerate recovery between training sessions, allowing for a more favorable adaptive response. This Spatiotemporal Collaborative optimizes phosphocreatine resynthesis and neural drive, significantly enhance the rate of force development (Sarfabadi et al., 2023).

### 2.4 Coherent adaptation hierarchy

The proposed fiber-type-specific hypertrophy mechanisms for the three BFRT modalities are synthesized in Figure 2. This figure serves as a graphical summary, hypothesizing the dominant adaptive pathways (fiber type recruitment) prioritized by each modality (BFR-LIRT, S-BFRRT, C-BFRRT), based on inferences drawn from the physiological mechanisms in the existing literature reviewed in the preceding sections. The adaptation continuum ranges from the metabolically dominant BFR-LIRT to the acute synergy of S-BFRRT and the chronic, integrated adaptations of C-BFRRT. While all modalities share core metabolic drivers (lactate/HIF-1α), their adaptive scope diverges, with BFR-LIRT prioritizing type I fibers through GH/IGF-1/mTOR signaling. S-BFRRT amplifies type IIa/x hypertrophy via mechanical-metabolic crosstalk. The C-BFRRT systematizes adaptations into angiogenic-neural loops for type IIx specialization. Hypertrophic efficacy varies by modality, with C-BFRRT inducing the greatest muscle hypertrophy (Scott et al., 2023; Wilk et al., 2018), These findings affirm the mechanistic reciprocity between metabolic stress and mechanical tension. Current evidence supports BFR-LIRT for tissue-sparing metabolic stimulus, S-BFRRT for concurrent strength-hypertrophy adaptation, and C-BFRRT for maximal morphological changes, providing clinicians and coaches with a mechanistic framework for evidence-based implementation of these methods.

## 3 Practical applications and advantages of different BFRT modalities

Table 1 synthesizes key findings from existing research on blood flow restriction resistance training (BFRT) and its effects on skeletal muscle strength and morphology in athletes. The studies included in this table were selected to provide a representative overview of the empirical evidence available for each modality. Selection was primarily based on the following criteria: (1) investigation in athletic or highly trained populations; (2) inclusion of a BFRT intervention group with clearly defined parameters (load, pressure, volume); and (3) measurement of relevant outcomes related to strength and/or morphological adaptations. This approach aimed to capture a range of evidence across different sports to illustrate practical applications, rather than to constitute an exhaustive systematic inventory. This study systematically compared different BFRT modalities (BFR-LIRT, S-BFRRT, and C-BFRRT), highlighting their respective training parameters and performance. The compiled evidence demonstrates that BFRT effectively enhances muscle hypertrophy, strength gains, and sport-specific performance. Additionally, it provides practical insights into optimal pressure application, exercise selection, and program design for athletes. These findings underscore the potential of BFRT as a valuable adjunct to traditional resistance training.

**TABLE 1:** Effects of blood flow restriction resistance training on skeletal muscle strength and morphology.

Exercise modality	Reference	Sport	(1) Volume and load	Exercise and duration	BFRT program	Performance	Main conclusions
			(2) Repetition tempo (ECC:CON)				
BFR-LIRT	Li et al. (2019)	Handball (n = 16)	(1) 4sets (30%1RM) 30–15–15–15 (60s) (2) NA	Squat and deadlift lunge 2×/wk. For 4 wk	(1) PC(5 cm) (2) Lower limb (3) 200 mmHg (4) IC	Thigh circumference *; Knee flexor MVC↑	BFR-LIRT demonstrates superior efficacy to HLRT in knee isokinetic strength and maximal force production
	Wang et al. (2019)	Handball (n = 18)	(1) 4sets (30%1RM) 30–20–20–20 (60s) (2) NA	Squat and deadlift lunge 3×/wk. For 8 wk	(1) PC(5 cm) (2) Lower limb (3) 200→220 mmHg (4) CC	Peak knee flexion torque↑; SJ and CMJ*	BFR-LIRT Outperforms HLRT in Hamstring Strength Development
	Ugur et al. (2023)	Canoe (n = 16)	(1) 3sets (30%1RM) 10–15reps (60s) (2) NA	Leg press 3×/wk. For 8 wk	(1) PC(7 cm) (2) Lower limb (3) 180→230 mmHg (4) NA	Rectus femoris CSA、Peak torque↑	BFR-LIRT achieves faster muscular hypertrophy and strength gains
	Yang et al. (2022)	Gymnastics (n = 15)	(1) 3sets (30%1RM) 10–12reps (90s) (2) NA	Back squat and front squat 2×/wk. For 4 wk	(1) EC (7.5 cm) (2) Lower limb (3) PPT (4) NA	Rectus femoris CSA、Isometric Strength*	BFR-LIRT shows comparable effectiveness to HLRT for lower strength improvement
	Korkmaz et al. (2022)	Football (n = 23)	(1) 4sets (30%1RM) 30–15–15–15 (30s) (2) NA	Knee extension and flexion 2×/wk. For 6 wk	(1) PC(7 cm) (2) Lower limb (3) 130→150 mmHg (4) NA	Knee joint MVC*; Rectus femoris CSA↑	BFR-LIRT shows superior hypertrophic effects vs. HLRT
	Abe et al. (2005)	Track and field (n = 15)	(1) 3sets (20%1RM) 15reps (30s) (2) NA	Squat and Leg Press 2×/d for 8 d	(1) PC(5 cm) (2) Lower limb (3) 160→240 mmHg (4) CC	Strength (Squat)、Rectus femoris CSA↑	BFR-LIRT is a highly effective method for enhancing muscle hypertrophy
	Wang et al. (2023)	Swimming (n = 16)	(1) 4sets (20%1RM) 30–15–15–15 (60s) (2) 1:1(s)	Squat 3×/wk. For 8 d	(1) PC(6 cm) (2) Lower limb (3) 200 mmHg (4) IC	Strength (Squat)*	BFR-LIRT and HLRT show similar strength improvement effects
S-BFRRT	Geng et al. (2021)	Alpine Skiing (n = 6)	(1) HLRT:3sets (75%1RM) 5–10reps; BFR-LIRT:4sets (30%1RM)30–20–20–20 (45s) (2) 1.5:1.5(s)	Leg Press and knee extension and flexion 4×/wk. For 2wk	(1) PC(5 cm) (2) Lower limb (3) 200→300 mmHg (4) NA	Rectus Femoris MVC、RMS↑	S-BFRRT outperforms HLRT in lower limb maximal strength and rate of force development
	Yamanaka et al. (2012)	Football (n = 32)	(1) HLRT:3sets (75%1RM)10reps BFR-LIRT:4sets (20%1RM) 30–20–20–20 (45s) (2) 2:1(s)	Bench press and Squat 3×/wk. For 4 wk	(1) EC (5 cm) (2) Upper and lower limbs (3) AOT (4) CC	Strength (bench press and Squat)、Rectus femoris CSA↑	S-BFRRT achieves faster strength gains than traditional training
	Luebbers et al. (2014)	Football (n = 16)	(1) HLRT:5sets (65%→90%1RM) (8-6-4-2-2) BFR-LIRT:4sets (20%1RM)30–20–20–20 (45s) (2) 1.5:1.5(s)	Bench press and Squat 4×/wk. For 7 wk	(1) EC (7.6 cm) (2) Upper and lower limbs (3) AOT (4) CC	Strength (Squat)、Rectus femoris CSA↑	S-BFRRT effectively enhances lower limb maximal strength
	Scott et al. (2017)	Football (n = 11)	(1) HLRT:3sets (80%1RM) 6–8 reps; BFR-LIRT:4sets (30%1RM) to failure (30s) (2) NA	Single-Leg deadlift 2×/wk. For 6 wk	(1) PC(17 cm) (2) Lower limb (3) 140 mmHg (4) CC	Knee joint isokinetic strength、Hamstring muscle CSV↑	S-BFRRT demonstrates superior efficacy over HLRT for lower limb maximal strength and hypertrophy
C-BFRRT	Che et al. (2022)	Wrestling (n = 16)	(1) HLRT:4sets (70%→75%1RM)8–10 reps (60s); BFR-LIRT:4sets (30%1RM)30–15–15–15 (60s) (2)NA	Bench press and deadlift 3×/wk. For 6 wk (H:2,B:1)	(1) PC(5 cm) (2) Lower limb (3) 180 mmHg (4) NA	Knee joint isokinetic strength↑	C-BFRRT demonstrates superior lower limb strength benefits compared to isolated HLRT or BFR-LIRT
	Zhu 2021	Taekwondo (n = 20)	HLRT:4 sets (70%→80%1RM10reps (90s; BFR-LIRT:4sets (20%→30%1RM)30–15–15–15 (90s) (2) 1.5:1.5 (s)	Squat、deadlift and lunge 3×/wk. For 8 wk (H; 1,B:2)	(1) PC(5 cm) (2) Lower limb (3) 200 mmHg (5) IC	Rectus femoris CSA、CMJ、SJ、Knee joint isokinetic strength↑	C-BFRRT induces superior muscle hypertrophy, endurance, and power adaptations
	Sarfabadi et al., 2023	Long jump (n = 16)	(1) HLRT:3 sets (60%→70%1RM)6-8reps; BFR-LIRT4sets (20%1RM) 30–15–15–15 (30s) (2) 2:1 (s)	Leg press 2×/wk. For 6 wk (H; 1,B:1)	(1) PC(17 cm) (2) Lower limb (3) 140 mmHg (4) CC	Strength (Squat)↑,Peak torque of quadriceps femoris *	C-BFRRT effectively enhances lower limb strength and explosive power

This table synthesizes data extracted directly from the cited original studies. Reps, repetitions; IC, Intermittent compression; CC, Continuous compression; EC, Elastic cuffs; PC, Pneumatic cuff; PPT, Perceived Pressure Technique; AOT, Absolute overlap technique; NA, Not applicable; MVC, Maximum voluntary contraction; CSV, Cross-sectional area; HLRT, High-load resistance training; CMJ, Counter movement jump; SJ, Squat jump; → Periodic increase; ↑ Significantly higher than the conventional group; * Significant within-group changes but no between-group differences; 20%–30%: numerical range; 75→ 90%: progressively increasing.

### 3.1 BFR-LIRT

Based on the empirical findings summarized in Table 1, blood flow restriction combined with low-load resistance training (BFR-LIRT) demonstrates superior efficacy compared with conventional resistance training. A recent meta-analysis (Su et al., 2025) revealed moderate improvements in muscle strength (ES = 0.65), providing robust evidence of the benefits of BFR-LIRT in athletes. Korkmaz et al. (2022) observed greater rectus femoris thickness and knee flexion/extension strength in soccer players using progressive pressure (130→150 mmHg), with improvements significantly surpassing high-load training outcomes (p < 0.05). Similarly, another study reported (Li et al., 2019) enhanced isokinetic knee strength and lower limb maximal strength after 4-week BFR-LIRT (200 mmHg, 30% 1RM). Ugur et al. (2023) reported a 16.2% strength improvement alongside muscle thickness gains in elite canoeists using incremental pressure (180→230 mmHg). Collectively, these findings suggest accelerated muscle adaptation through metabolic stress and cell swelling. This supports the view that BFR-LIRT resulted in greater muscle mass accumulation, highlighting its unique advantage in promoting muscle growth. BFR-LIRT has distinct advantages in athletic rehabilitation. Evidence from Lubowitz et al. (2022) demonstrated that 20% 1RM BFR-LIRT enhances functional recovery and reduces pain in athletes with talar fractures. For athletes with patellar tendinopathy, this intervention increased tendon thickness by 8.3% and lower extremity strength by 15.4% (Cuddeford and Brumitt, 2020) with marked improvements in VISA-P scores. These outcomes establish BFR-LIRT as an effective alternative for pain-restricted athletes, offering three key benefits: minimizing joint stress through low mechanical loads, maintaining anabolic stimulation via metabolic stress, and promoting tissue repair through enhanced local circulation.

Current evidence on BFR-LIRT shows notable heterogeneity. One study (Laurentino et al., 2012) found no significant effect on knee extensor stiffness with constant-pressure protocols (20% 1RM), while another study reported (Mitchell et al., 2019) inferior muscle thickness gains compared to high-load training after 5-week BFR-LIRT (110 mmHg). The divergent efficacy outcomes across BFR-LIRT studies may be attributed to three key methodological variables: (1) nonprogressive pressure application: fixed pressures (e.g., 110 mmHg; Martín-Hernández et al., 2013) fail to accommodate adaptive increases in limb circumference and arterial flow; studies implementing pressure progression (e.g., 180→230 mmHg; (Ugur et al., 2023)) have demonstrated greater hypertrophy, likely due to sustained metabolic challenge and type II fiber recruitment (Counts et al., 2016; Hanke et al., 2020). (2) Interindividual AOP variability: Prescribing absolute pressures (mmHg) ignores differences in arterial occlusion pressure (AOP), which varies by 40%–80% between athletes owing to limb composition and training status (Patterson et al., 2019). Relative pressures (50%–80% AOP) optimize stimulus individualization, as evidenced by 23% higher strength gains compared to fixed-pressure protocols (Zhang, Gao, et al., 2025). (3) Inadequate intervention duration: Adaptations require 4–6 weeks to achieve measurable hypertrophy (Luebbers et al., 2024). Shorter interventions (≤3 weeks) primarily elicit neural adaptations, which explains the null morphological findings (Laurentino et al., 2012). We hypothesize that optimizing BFR-LIRT requires: (1) pressure titration based on %AOP, (2) progressive overload (increasing pressure or volume by 10%–15% weekly), and (3) a minimum 4-week intervention to exploit metabolic and swelling synergy. While BFR-LIRT offers advantages for rehabilitation and tissue-sparing maintenance due to its low mechanical stress, its reliance on metabolic pathways alone may limit maximal strength and power development compared with protocols incorporating higher mechanical tension.

### 3.2 S-BFRRT

Unlike BFR-LIRT, which relies solely on metabolic stress, Supplementary blood flow restriction resistance training integrates high-load resistance exercise with subsequent low-load BFRT (1-2 exercises per muscle group) to optimize muscular adaptations through combined mechanical tension and metabolic stress. This approach effectively recruits typically under activated type I muscle fibers (Yasuda et al., 2011), as demonstrated by significant vastus lateralis hypertrophy in national weightlifters following 6-week S-BFRRT (30% 1RM squats post HLRT) (Bjornsen and Wernbom, 2019b). Supporting evidence from Paralympic skiers shows that 2-week high-frequency S-BFRRT (200*→*300 mmHg, 30% 1RM) preferentially improved weaker-leg MVC (p < 0.05), with female athletes exhibiting greater neuromuscular enhancements (RMS and RFD) than males (Geng et al., 2021). Yamanaka et al. (2012) found that 4-week S-BFRRT improved strength (bench press +7.0%, squat +8.0%) without limb hypertrophy, whereas Luebbers et al. (2014) later reported significant squat 1RM gains (p < 0.05) after 7 weeks. However, later findings (Scott et al., 2017) indicated no additional benefits from 5-week S-BFRRT during preseason training, potentially due to training saturation or fatigue. Collectively, these findings demonstrate the context-dependent efficacy of S-BFRRT, which is particularly valuable for rehabilitation, neuromuscular compensation, and in-season strength development when implemented with progressive pressure (150*→*300 mmHg) and a moderate load (30% 1RM). However, the current limitations, including small sample sizes and uncontrolled confounders, warrant further investigations.

Compared to BFR-LIRT, which primarily relies on metabolic stress for adaptation with minimal mechanical load, S-BFRRT leverages the synergy of high mechanical tension followed by amplified metabolic stress within one session. This dual-phase stimulus is uniquely suited for competitive phases in which concurrent strength and hypertrophy gains are prioritized, and frequent high-load sessions may interfere with sport-specific training. Unlike C-BFRRT, which distributes different stimuli across separate weekly sessions, S-BFRRT delivers a combined mechano-metabolic stimulus acutely.

### 3.3 C-BFRRT

Distinct from both BFR-LIRT (solely metabolic focus) and S-BFRRT (single-session combination), C-BFRRT is a periodic training method that combines HLRT with blood flow restriction resistance training in a specific proportion (e.g., two HLRT + one BFR session per week). Evidence from a previous study (Che et al., 2022) demonstrated superior gains in maximal strength and isokinetic knee strength in female wrestlers after 6-week C-BFRRT (180 mmHg, 30% 1RM) compared to conventional training. Subsequent research (Sarfabadi et al., 2023) reported greater lower-body strength and hypertrophy in long jumpers following 6-week C-BFRRT (140 mmHg, 20% 1RM) compared to high-load training alone. Further investigation (Zhu, 2021) confirmed these findings in taekwondo athletes, with C-BFRRT (200 mmHg, 20%–30% 1RM) producing superior muscular adaptation.

Current evidence preliminarily supports C-BFRRT as an effective training strategy, particularly during preparatory phases aimed at maximizing muscle hypertrophy (especially in type II fibers) and strength. Its core advantage lies in the spatiotemporal integration of mechanical and metabolic stimuli achieved through the periodized alternation of high-load sessions (which generate significant mechanical tension, microdamage, and satellite cell activation) and low-load BFRT phases (which prolong the anabolic window and enhance metabolic stress and cellular swelling). This unique combination endows C-BFRRT with the potential to induce superior morphological adaptations compared to isolated HLRT or BFR-LIRT modes (Che et al., 2022; Sarfabadi et al., 2023; Zhu, 2021). However, it must be emphasized that the current body of high-quality research specifically on the C-BFRRT modality remains limited (with only a few studies identified in this review), and significant heterogeneity in outcomes has not yet been reported. Future research requires more rigorously designed studies with larger sample sizes to validate its efficacy across diverse sports and populations (e.g., males vs. females, varying training backgrounds), assess long-term benefits, and further explore optimal implementation parameters (such as the specific ratio of high-to-low loads and alternation frequency).

## 4 Designing BFRT programs

This section translates the mechanistic insights and empirical findings discussed previously into evidence-informed practical guidelines. Table 2 serves as the central reference, synthesizing recommended training parameters for the three BFRT modalities. These recommendations are the product of a qualitative synthesis of studies identified through targeted searches of academic databases (e.g., PubMed, Google Scholar) using keywords related to BFRT, athletes, strength, and hypertrophy. Priority was given to randomized controlled trials, longitudinal studies, and high-quality case studies conducted in athletic populations. The primary objective of these distinct approaches is to achieve specific adaptive outcomes: BFR-LIRT aims for rehabilitation and tissue-sparing maintenance, S-BFRRT for concurrent strength and hypertrophy during demanding phases, and C-BFRRT for maximal morphological adaptations during dedicated preparation periods. The following subsections (4.1–4.8) provide a detailed rationale and discussion for the parameters summarized in Table 2.

**TABLE 2:** Recommendations for BFR resistance training for athletes based on relevant studies.

Category	BFR-LIRT	S-BFRRT	C-BFRRT
Training parameters			
Load	20%–30% 1RM	HLRT: 75→90%1RM + BFRT: 20%–30%1RM	Periodized: 70→85% ↔ 20%–30%1RM
Volume	4 sets (30–15–15–15 reps)	HLRT: (3−5 sets)×(5−10 reps) + BFRT: 4 sets×(30–20–20–20 reps)	HLRT: 3 sets × (6–8 reps) + BFRT: 4sets×(30–15–15–15 reps)
Frequency	2–3 sessions/week	3–4 sessions/week	HLRT:2–3 + BFRT:1–2 sessions/week
Rest Intervals	30–60s	45–60s	30–90s
Tempo	Not specified (NA)	E:C 1.5:1.5–2:1s	E:C1:1–1.5:1.5s
Exercise Type	Multi-joint or single-joint	Combining both	Combining both
BFRT Parameters			
Pressure	40→80% AOP(Biweekly increase ≤10%)		
Cumulative BFRT Duration	Total time:<30 min upper limbs:15–20 min lower limbs:20–30min		
Clinical Applications	• Postoperative rehab • In-season maintenance	• Strength-power athletes • Concurrent training phases	• Hypertrophy-focused athletes • Off-season strength building
Adaptation Timeline	4–6 weeks	6–8 weeks	8–12 weeks

These recommendations are based on the authors’ synthesis of the existing evidence presented in this review and should be interpreted as practical guidelines rather than unequivocal prescriptions; E:C, Eccentric:Concentric; 20%–30%: numerical range; 75→ 90%: progressively increasing.

### 4.1 Exercise load

A comprehensive analysis of BFRT parameters revealed that low-load BFR-LIRT (20%–50% of 1RM or 20%–50% MVC) elicits muscular hypertrophy comparable to traditional HLRT while maintaining superior safety and reduced joint stress (Hanke et al., 2020). The most effective load range appears to be 20%–50% 1RM, as loads below 20% demonstrate diminished hypertrophic effects, and those exceeding 50% provide no additional benefits despite increased discomfort. The progression of load in BFR resistance training remains controversial in the sports science literature. Current evidence suggests differential approaches based on training modality and athletic population.

For BFR-LIRT, maintaining 20%–30% 1RM throughout the cycle appears optimal, as evidenced by a study (Li et al., 2019) that showed consistent strength gains (10%–15% isokinetic torque) without load progression in handball players. The metabolic stress mechanism (lactate ↑2.5–3.8 mmol/L) dominates the adaptation pathways at these loads, making absolute load increases unnecessary. Conversely, S-BFRRT may benefit from progressive overload in the high-load component; a previous study (Luebbers et al., 2014) reported superior outcomes when increasing HLRT loads from 65% to 90% 1RM over 7 weeks while keeping BFRT bouts at 20% 1RM, likely due to the preserved contrast of the metabolic-mechanical stimulus. Current data support alternating and progressive intensities for C-BFRRT. Che et al. (2022) found that alternating HLRT (70→85% 1RM) with BFRT (20%–30% 1RM) yielded greater muscle maximal strength gains *versus* linear progression in wrestlers. This periodization strategy enhances both mTOR phosphorylation and type II fiber hypertrophy, suggesting that mechanical tension remains crucial in hybrid protocols.

### 4.2 Training volume, frequency and recovery

Regarding set and repetition schemes, two primary protocols have emerged as effective: the 30/15/15/15 protocol (total 75 reps) and the 4× (15–20 reps) protocol (total 60–80 reps), both of which are typically performed to volitional fatigue (Rolnick and Kamis, 2023; Wang et al., 2022). Current research recommends relatively short rest intervals (30–90 s) between sets to maintain metabolic stress while allowing sufficient recovery for subsequent sets (Ugur et al., 2023; Yang et al., 2022). For training frequency, 2–3 sessions per week appears optimal, with studies showing significant morphological adaptations after only 3 weeks of consistent training (Bjornsen and Wernbom, 2019a). The minimum effective dose for measurable hypertrophy is approximately 12 sessions (3 weeks at 4 sessions/week or 4 weeks at 3 sessions/week) (Luebbers et al., 2024).

### 4.3 Repetition tempo

Repetition tempo optimization in blood flow restriction resistance training should be precisely tailored to specific training objectives. For hypertrophy-oriented training, a controlled tempo of 3–4 s for the eccentric (ECC) phase combined with 1–2 s for the concentric (CON) phase is recommended to maximize the time under tension and metabolic stress, thereby promoting muscular growth (Centner et al., 2019). When targeting strength and power development, a faster tempo of 1–2 s for the ECC phase with explosive CON contractions (<1 s) is more effective for enhancing neuromuscular recruitment and type II fiber activation (Counts et al., 2016). Rehabilitation and endurance applications benefit from slower tempos (4–6 s for the ECC phase with 2 s for the CON phase) to minimize joint loading while preventing premature fatigue. Critical considerations include avoiding excessive ECC durations (>6 s), which may compromise the technique or induce ischemic fatigue, and ensuring that the CON velocity is sufficient to maintain the benefits of neural adaptation (Korkmaz et al., 2022). Optimal tempo selection must integrate three key factors: (1) primary training goals (hypertrophy/power/rehabilitation), (2) exercise characteristics (single/multi-joint), and (3) individual athlete factors (training status and joint integrity) to ensure both efficacy and safety.

### 4.4 Single-joint vs. multi-joint exercises

Exercise selection for BFRT depends on training goals and individual adaptability. Both single-joint (e.g., leg extension) and multi-joint (e.g., squat) exercises effectively promote muscular adaptations during BFRT. Single-joint movements are suitable for rehabilitation and isolated muscle training because of their precise targeting (Diao et al., 2025). Multi-joint exercises enhance functional capacity and systemic strength through increased muscle recruitment (Pope et al., 2013). Training goals dictate the selection of exercises: rehabilitation favors single-joint exercises, whereas functional training prioritizes multi-joint movements. Combining both (e.g., BFRT squats + leg extensions) provides comprehensive benefits (Huang and Park, 2024) but requires an adjusted cuff pressure and programming (Slysz et al., 2016).

### 4.5 Pressure progression

The progression of occlusion pressure in BFRT remains debatable, with current evidence suggesting context-dependent strategies. For athletic populations, pressure prescription should consider limb composition, training phase, and individual arterial occlusion pressure (AOP). Recent studies have demonstrated that fixed relative pressures (40%–80% AOP) maintain efficacy throughout 6–8-week cycles without requiring progressive increases, as metabolic stress mechanisms (lactate accumulation and cellular swelling) remain effective at consistent pressures (Patterson et al., 2019). However, Evidence in reference (Hanke et al., 2020) reported greater strength gains when progressively increasing pressure (50→70% AOP) in resistance-trained athletes, likely due to enhanced type II fiber recruitment under escalating metabolic challenge.

Practical considerations for pressure progression in BFRT should account for: (1) limb circumference changes, requiring periodic pressure adjustments to maintain relative occlusion as muscle mass increases (Huang and Park, 2024); (2) training phase specificity, where higher pressures (70%–80% AOP) optimize hypertrophy phases, whereas lower pressures (40%–50% AOP) are more suitable for reloading periods; and (3) individual tolerance, with gradual pressure increases (∼10% every 2 weeks) demonstrating improved compliance in novice athletes (Zhang and Xu, 2025). Notably, excessive pressure progression (>80% AOP) may impair technical execution and increase the risk of thrombosis without augmenting adaptations (Su et al., 2025). Current evidence supports individualized pressure titration over rigid progression models, particularly when combined with load progression in hybrid protocols.

### 4.6 Cumulative BFRT duration

For athlete BFRT, the total occlusion time per session should be 15–30 min, including intermittent pressure release between the sets. Upper-limb BFRT typically requires shorter durations than lower limbs. For lower limb training, a total occlusion time of 20–30 min is recommended; exceeding 30 min may increase the risk of venous thrombosis. Upper limb sessions should limit occlusion to 15–20 min, with continuous single-limb occlusion not exceeding 10 min of occlusion. The total session occlusion must remain under 30 min. Pressure should be immediately released if paresthesia occurs (Wortman et al., 2021).

### 4.7 Intermittent vs. continuous pressure

The application of BFRT during resistance training presents distinct physiological and practical implications when intermittent and continuous protocols are compared. Intermittent BFRT (typically employing 30–60s occlusion/30s release cycles) demonstrates superior safety characteristics, with evidence from a previous study (Wang et al., 2022) documenting significantly lower vascular stiffness markers in athletes than in those with continuous application. This protocol effectively maintains metabolic stress (lactate >8 mmol/L) while permitting partial metabolite clearance, thereby reducing the perceptual discomfort. In contrast, continuous BFRT induces more pronounced cellular swelling and enhanced acute mTOR phosphorylation responses (Wang et al., 2023).

For athletic implementation, protocol selection should be guided by specific training objectives and environmental factors of the sport. Intermittent BFRT is particularly effective for (1) technical skill integration during sport-specific movements, (2) extended training sessions (>30 min), and (3) heat-acclimated environments where periodic pressure release mitigates cardiovascular strain (Scott et al., 2023). Conversely, continuous BFRT offers distinct advantages for (1) brief hypertrophy-focused sessions (≤20 min) and (2) post-activation potentiation strategies (Zheng et al., 2022). This dichotomy reflects the fundamental trade-off between the superior safety profile of intermittent BFRT and the enhanced metabolic and mechanical stimuli of continuous BFRT.

Notable performance outcomes include findings from (Smith et al., 2022), who reported 4.1% greater VO2max improvements with intermittent BFRT in endurance athletes *versus* 6.3% CMJ enhancement with continuous protocols in power athletes. Practical implementation considerations include equipment constraints, with pneumatic systems facilitating intermittent cycling, whereas elastic wraps typically require continuous application. Emerging hybrid models (e.g., 5:2 min intermittent: continuous cycles) show promise for optimizing both safety and efficacy, although they require further investigation (Su et al., 2025).

### 4.8 Periodized training

**FIGURE 3:**
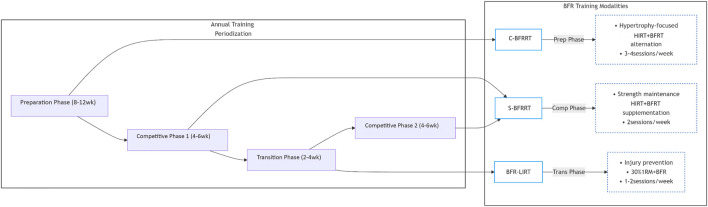
Application of different BFRT modes in annual cycle.

The periodized application of BFRT modalities follows a logical temporal sequence that is aligned with specific training objectives. During the 8–12 weeks preparation phase, C-BFRRT optimally induces hypertrophy by alternating high-load mechanical tension and metabolic stress stimuli. As athletes transition into the 4–6 weeks competitive phase, S-BFRRT effectively maintains neuromuscular performance by preserving mTOR activation and type II fiber recruitment, despite reduced training volumes. Finally, during the 2–4 weeks transition phase, BFR-LIRT facilitates active recovery by enhancing metabolic clearance while maintaining protein synthesis. This systematic approach ensures biological synchronization between the imposed training stimuli and desired physiological adaptations while respecting the minimum effective durations required for each modality (8, 6, and 4 weeks for C-BFRRT, S-BFRRT, and BFR-LIRT, respectively). Figure 3 conceptualizes this periodized application, aligning each BFRT modality with the specific objectives of different training phases within an annual cycle (preparatory, competitive, transition). It should be interpreted as a proposed model for integration based on the theoretical rationale and limited empirical evidence available, rather than a universally validated prescription.

The periodized application of BFRT modalities follows a logical temporal sequence aligned with specific training objectives. Taking the annual training cycle of basketball players as an example, during the 8–12 weeks preparation phase, athletes focus on muscle hypertrophy and foundational strength development by adopting the C-BFRRT modality. This involves alternating high-intensity training sessions (e.g., squats and deadlifts at 70%–85% 1RM) with low-intensity BFRT sessions (e.g., leg press at 30% 1RM) on a weekly basis, effectively activating type II muscle fibers and increasing muscle cross-sectional area. Upon entering Competition Phase 1 (4–6 weeks), the training emphasis shifts to maintaining maximal strength, making S-BFRRT the preferred modality. For instance, after technical training during game weeks, high-intensity bench presses (90% 1RM) combined with low-load BFR bench presses (20% 1RM) can be implemented to prolong mTOR pathway activation and preserve type IIa/x muscle fiber function. If the season comprises multiple segments (e.g., regular season and playoffs), S-BFRRT may continue to be used in Competition Phase 2, with a transition to BFR-LIRT during the interval between competitive periods to maintain muscle metabolic activity with low joint load, thereby preserving strength while minimizing excessive fatigue. During the transition phase (2–4 weeks) after the season, BFR-LIRT is prioritized, involving 1–2 weekly sessions of full-body low-intensity circuit training (30% 1RM) to promote neuromuscular recovery and injury repair while sustaining basal anabolic levels.

## 5 Conclusion

This review synthesizes the current literature to compare the theoretical mechanisms and practical applications of three primary BFRT modalities (BFR-LIRT, S-BFRRT, C-BFRRT). Based on a narrative synthesis of available studies, it appears that BFRT can achieve unique physiological effects by modulating mechanical tension and metabolic stress pathways to varying degrees. The evidence suggests that BFR-LIRT may primarily induce type I fiber hypertrophy through metabolic stress, making it a valuable tool for rehabilitation. S-BFRRT seems to create acute synergy by combining high-load tension with metabolic stimuli, potentially benefiting strength maintenance. C-BFRRT, through its periodized structure, holds promise for inducing integrated adaptations, potentially favoring type II fiber specialization during preparatory phases.

However, these conclusions must be interpreted with caution due to several key limitations. The current body of evidence is characterized by studies with small sample sizes, significant methodological heterogeneity (e.g., in pressure application protocols, exercise selection, and intervention duration), and a lack of direct comparative meta-analyses. Furthermore, important safety considerations, particularly regarding thrombosis risk in athletes with potential hypercoagulable states and the long-term effects of repetitive BFRT, remain underexplored and warrant careful attention in practice.

Therefore, the proposed hierarchical system for targeting fiber-type-specific adaptations across training phases should be viewed as a preliminary framework grounded in mechanistic rationale and emerging empirical evidence, rather than a definitive prescription. Future high-quality research with larger samples, standardized protocols, longer follow-ups, and direct comparative designs is urgently needed to validate these findings, establish clearer efficacy hierarchies, and elucidate optimal implementation parameters. Prioritizing safety and individualized application remain paramount.
